# Enhancing Advance Care Planning Engagement: Insights from Cultural and Family‐Based Interventions for Asian and Hispanic American Dementia Caregivers

**DOI:** 10.1002/alz70858_103980

**Published:** 2025-12-26

**Authors:** Jung‐Ah Lee, Hyun Jung Kim

**Affiliations:** ^1^ University of California, Irvine, Irvine, CA, USA; ^2^ Seattle University, Seattle, WA, USA

## Abstract

**Background:**

The importance of early and ongoing Advance Care Planning (ACP) discussions in dementia care has been emphasized due to dementia's progressive nature. While its benefits are well documented, the acceptance and delivery of ACP discussions require consideration of cultural and social factors that influence future care, decision‐making, and end‐of‐life preferences for people with dementia (PWD) and their families.

**Methods:**

This descriptive qualitative study examined reports from Asian and Hispanic American caregivers about their caregiving experiences to identify important ACP components for these communities. Thematic analysis was performed using home‐visit logs from a randomized controlled trial (RCT) designed to support dementia family caregivers in the United States. Participants recruited from ethnic communities received weekly education sessions led by trained bilingual community home visitors, focusing on caregiving skills, including ACP.

**Results:**

A total of 115 Asian and Hispanic American caregivers participated in the RCT. About 60% were adult offspring, and the rest were spousal caregivers of PWD. Key findings from the thematic analysis regarding the experiences of caregivers in ACP included legal and financial planning, family involvement in decision‐making, cultural attitudes toward ACP, and proactive health and safety measures. Asian and Hispanic caregivers discussed legal and financial planning as a significant concern, struggling with initiating discussions about advance directives and other formal preparations. Challenges in family involvement in decision‐making revealed issues such as sibling disagreements, uneven caregiving responsibilities, and emotional conflicts over end‐of‐life care. Cultural attitudes toward ACP revealed hesitancy among Asian and Hispanic caregivers, influenced by reluctance to discuss topics such as death or incapacity. Lastly, proactive health and safety measures indicated caregivers’ efforts to ensure safety and structured routines to promote their PWD's well‐being.

**Conclusions:**

The study underscores the need for interventions that address cultural stigma and hesitancy surrounding ACP discussions while promoting accessible, linguistically appropriate resources. Family‐centered strategies encouraging shared caregiving responsibilities and reducing emotional conflicts are crucial for fostering collaborative decision‐making. Future research should examine the long‐term impact of these interventions on caregiver well‐being and ACP implementation.

